# Editorial: Gut Microbiota and Inflammation: Relevance in Cancer and Cardiovascular Disease

**DOI:** 10.3389/fphar.2020.613511

**Published:** 2020-12-23

**Authors:** Cinzia Parolini, Amedeo Amedei

**Affiliations:** ^1^ Department of Pharmacological and Biomolecular Sciences, Università degli Studi di Milano, Milan, Italy; ^2^ Department of Experimental and Clinical Medicine, University of Florence, Florence, Italy; ^3^ SOD of Interdisciplinary Internal Medicine, Azienda Ospedaliera Universitaria Careggi (AOUC), Florence, Italy

**Keywords:** gut microbiota, cancer, cardiovasclar disease, low density lipoprotein—cholesterol, calcific aortic valve disease

**Editorial on the Research Topic**

**Gut Microbiota and Inflammation: Relevance in Cancer and Cardiovascular Disease**

The chronic inflammatory process is the mutual starting factor of numerous non-communicable diseases, including cancer and cardiovascular, neurologic, respiratory, and metabolic disorders. Directly or indirectly, the inflammatory mediators can favor the cancer development, inducing mutations in different loci, especially in tumor-suppressor genes, or inferring with post-translational modifications and favoring aberrant DNA methylation.

Increasing and recent evidence link the mammal inflammatory status and correlated immune response with the microbiota, a complex ecosystem of microorganisms, such as bacteria, archaea, fungi, viruses, and protozoans (Amedei, 2019; Boem et al., 2020; Niccolai et al., 2020). The gastrointestinal tract shows the greatest microbial diversity and density, named gut microbiota (GM), that exerts crucial nutritional (e.g., carbohydrates fermentation) and metabolic functions, including the xenobiotic metabolism (Rowland et al., 2018). In addition, GM is essential for the correct development of the gut-associated lymphoid tissue (GALT) and the regular evolution of the innate and specific immune system (Cebra, 1999). Instead, the immune system itself has evolutionarily developed to favor a symbiotic relationship with different microorganisms (Belkaid and Hand, 2014).

Having made these premises, the aim of our research topic has been to gather new information regarding the mechanisms regulating the early phases of these disabling inflammation-based human diseases.

In eubiosis, the microbiota-immunity axis facilitates the ideal orchestration of both innate and adaptive immune response in order to modulate the most appropriate reaction (Zitvogel et al., 2016). In contrast, dysbiosis can change the immune status, rendering the host susceptible to endo/exogenous alterations, breaking the tolerance vs. self-components, and triggering immune responses deficient or excessive (the so-named chronic inflammation). In turn, this might support the beginning of cancer and autoimmune diseases, and the GM could represent a non-negligible link between these two contrasting disorders.

In line with this, Cao et al. explored the hypothesis that *Fusobacterium nucleatum*) could intensify the intestinal inflammation, promoting the intestinal mucosal barrier damage. In fact, increasing data suggest that GM bacteria, especially *F. nucleatum*, are associated with Crohn’s disease (CD), but it is not clear how the *F. nucleatum* supports the CD pathogenesis. The authors documented that *F. nucleatum* was enriched in 41.21% of CD tissues and, of note, was correlated with the clinical course and activity, and refractory CD behavior. In addition, they showed that *F. nucleatum* infection is linked to the activation of the endoplasmic reticulum stress (ERS) pathway in the CD progression to promote the devastation of the intestinal mucosal barrier. To define the specific mechanisms involved, they used *in vitro* (human normal epithelial cell line NCM460 and the FHC cell line ATCC) and the mouse model of Crohn’s disease and showed that the *F. nucleatum* targeted caspase activation and recruitment domain 3 (CARD3) to activate the ERS pathway and promote *F. nucleatum–*mediated mucosal barrier damage. Therefore, *F. nucleatum* coordinates a molecular network involving CARD3 and ERS to control the CD process. In conclusion, the authors suggest that quantifying and targeting *F. nucleatum* (and its associated pathways) could provide valuable insight into both the prevention and treatment of Crohn’s disease.

As previously reported, and recently well-reviewed (Novakovic et al., 2020; Oikonomou et al., 2020), different studies link the interplay microbiota-immune response with cardiovascular diseases, for which hypertension represents the main risk factor. Hypertension is a clinical condition, resulting from a multifaceted interplay of endogenous and environmental factors, where the GM role has been strongly supposed but is still poorly understood. To clarify this scenario, Silveira-Nunes et al. evaluated the fecal microbiota of hypertensive patients, investigating at the same time the serum cytokines’ signature as immunological profile.

In the enrolled patients, they documented a clear intestinal dysbiosis characterized by reduced biodiversity compared with the normotensive counterpart. In fact, along with a reduction in *Bacteroidetes* members, hypertensive patients showed an increased number of *Lactobacillus* and *Akkermansia* and decreased relative abundances of butyrate-producing bacteria, such as *Roseburia* and *Faecalibacterium,* within the Lachnospiraceae and Ruminococcaceae families. In addition, in the same hypertensive patients, the authors documented an altered inflamed status characterized by an amplified TNF and IL-6 production and increased TNF/IFN-γ ratio. In other words, Silveira‐Nunes et al. document, for the first time, an evident association of hypertension with altered GM composition and inflammation status pointing to ignored bacteria as potential contributors to intestinal homeostasis loss and thus the high vulnerability of hypertensive patients to inflammation-related pathologies.

Several studies have shown that the development of atherosclerosis, the dominant cause of cardiovascular diseases, is associated with trimethylamine N-oxide (TMAO) levels (Koeth et al., 2013; Illiano et al., 2020). Specifically, Yang et al. reported how TMAO may affect inflammation, immune response, and cholesterol metabolism as well as atherothrombosis processes, and therefore the development of atherosclerosis. In addition, the authors described clinical studies demonstrating that increased plasma levels of TMAO are a risk factor for i) major adverse cardiovascular events (stroke, myocardial infarction, or death) in atherosclerotic patients and ii) subsequent cardiovascular events among patients with recent prior ischemic stroke (Tang et al., 2013; Haghikia et al., 2018). The gut microbiota is one of the crucial factors in the TMAO generation, and changes in its composition have marked effects on TMAO levels. Indeed, Yang et al. reported that the administration of *Lactobacillus plantarum* ZDY04, *Enterobacter aerogenes* ZDY01, and *Enterococcus faecium* WEFA23 decreased serum TMAO concentrations by remodeling gut microbiota in mice. The authors suggest that TMAO could be used as a novel approach for the prevention and treatment of atherosclerosis.

In line with these data, Alushi et al. describe the potential relationship between gut microbiota metabolites, including TMAO, and the calcification of the aortic valve. The authors reviewed the calcific aortic valve disease (CAVD) pathophysiology, highlighting that inflammation and immune system activation are the common factors between this disease and atherosclerosis (Hulin et al., 2018). They hypothesize a role of the microbiota in the development and progression of CAVD, through a direct valvular damage caused by specific bacterial taxa or a stimulation of immune response and valve calcification. The former mechanism seems to be related to the oral microbiota because increasing evidence supports the presence of oral bacteria in the valvular tissue, and it has been demonstrated that specific strains of *Streptococcus* mutants have selective virulence for infectious endocarditis (Cohen et al., 2004). For the second indirect mechanism, as stated above, TMAO levels appear to be related to calcification degree, whereas short-chain fatty acids (generated by bacterial fermentation of dietary fiber) seem to promote the shifting of the immune response between pro- and anti-inflammatory pathways (Russo et al., 2016). Altogether, these data suggest that the microbiota could play a role in the development of CAVD.

Of note, Mendelian randomization analyses have strengthened the linear correlation between the concentration of low-density lipoprotein cholesterol (LDL-C) and the incidence of cardiovascular events. The LDL-C concentration has been identified as a primary causative and modifiable risk factor for the development of atherosclerosis (Parolini, 2020). The review by Villette et al. describe the gut microbiota’s impact on cholesterol levels. Recent data from epidemiological studies reported associations between phylum, bacteria taxa, and cholesterolemia. Bile acid biosynthesis is the predominant metabolic pathway for cholesterol catabolism in the human body (Hofmann, 1999); however, cholesterol is actively metabolized by intestinal bacteria, mainly in coprostanol, which is very poorly absorbed by the intestine (Gérard, 2013). The intestinal bacteria are also responsible for the conversion of primary bile acids to secondary bile acids (Gérard, 2013). In humans, antibiotic treatment causes cholesterol reduction mainly through the inhibition of the above-mentioned process determining a decrease in the hydrophobicity of bile acids. Interestingly, gut microbiota can affect the efficacy of statins, the leading pharmaceutical class in hyperlipemia therapeutic care.

In summary, the papers contained in this special issue provide new data supporting the role played by the intestinal microbiota in Crohn’s disease and hypertension, as well as in the development of atherosclerosis, CAV disease, and serum cholesterol levels.

## Author Contributions

The authors have made a substantial, direct and intellectual contribution to the work, and approved it for publication.

## Conflict of Interest

The authors declare that the research was conducted in the absence of any commercial or financial relationships that could be construed as a potential conflict of interest.
